# New Insights into the Applications of Viruses to Biotechnology

**DOI:** 10.3390/v15122322

**Published:** 2023-11-26

**Authors:** Patrick Materatski, Carla Varanda

**Affiliations:** 1 MED—Mediterranean Institute for Agriculture, Environment and Development & CHANGE—Global Change and Sustainability Institute, Institute for Advanced Studies and Research, Universidade de Évora, Pólo da Mitra, Ap. 94, 7006-554 Évora, Portugal; 2 CERNAS—Research Centre for Natural Resources, Environment and Society, ESAS - Escola Superior Agrária de Santarém Instituto Politécnico de Santarém, Quinta do Galinheiro, S. Pedro, 2001-904 Santarem, Portugal

## 1. Introduction

Viruses are responsible for many devastating human and animal diseases, such as Ebola, rabies, HIV, smallpox, influenza, dengue, and SARS-CoV-2 [1]. The deleterious impact of viruses is also shown in agriculture, wherein plant virus diseases account for USD30 billion in annual losses globally, thereby threatening world food security [2].

Viruses are obligate parasites, and are present in all habitats where there is life. They are the most abundant agents on Earth; the estimated number of viral particles on earth is close to 10^31^ [3,4]. Needless to say, the vast majority of viruses are unknown; however, the breakthrough and development of new sequencing technologies such as next-generation sequencing are accelerating the discovery of new viruses [5]. The world of technology is evolving at a rapid pace, and innovative technologies are continuously being discovered, allowing the genetic manipulation of these available simple viral systems and making them attractive tools for exploitation in different fields of science.

We can go back to the 18th century to find the first application of viruses in biotechnology; when, cowpox pustules were used to combat smallpox [6]. All subsequent scientific advances have enabled us today to use highly efficient vaccines containing genetically modified viruses to combat COVID-19 [7].

Viruses are being used in the prevention and treatment of many infectious diseases or cancer, not only through vaccines, but also as vectors to carry and deliver substances in situ, in which we can take advantage of their capacity to target specific cells [8]. In agriculture, viruses have been studied to introduce desirable characteristics in plants, showing their potential in plant breeding and plant protection [9]. Viruses have also been used in materials science and nanotechnology as a source of nanoparticles and as building blocks [10]. Other industries such as pharmacology, cosmetics, electronics, are some areas that are also benefiting from the potential uses of viruses [11].

We cannot ignore the harm viruses can cause to us, but we also cannot ignore the good they can do, and the potential they have alongside the cutting edge technologies we possess. It is clear that the more they are studied, the more possibilities they offer.

In this Special Issue ‘The Application of Viruses to Biotechnology, 2022’, we have gathered up-to-date research on the use of viruses in biotechnology, reinforcing the contribution these extraordinary agents to significant advances in science that would not be possible without their existence.

## 2. Special Issue Overview

With regard to the success of our previous Special Issue “The Application of Viruses to Biotechnology”, in which 16 articles from the years 2020 and 2021 were collated (presenting, until this date, over 200 citations), we present a continuation of this theme in a second SI edition—“The Application of Viruses to Biotechnology, 2022”. This edition collates eight articles that show the most up-to-date research using viruses in biotechnology. These eight contributions are listed below.

1. de Moor, W.R.J.; Williamson, A.L.; Schäfer, G.; Douglass, N.; Gers, S.; Sutherland, A.D.; Blumenthal, M.J.; Margolin, E.; Shaw, M.L.; Preiser, W.; et al. LSDV-Vectored SARS-CoV-2 S and N Vaccine Protects against Severe Clinical Disease in Hamsters. Viruses 2023, 15, doi:10.3390/v15071409.
2. Yip, M.; Chen, J.; Zhi, Y.; Tran, N.T.; Namkung, S.; Pastor, E.; Gao, G.; Tai, P.W.L. Querying Recombination Junctions of Replication-Competent Adeno-Associated Viruses in Gene Therapy Vector Preparations with Single Molecule, Real-Time Sequencing. Viruses 2023, 15, doi:10.3390/v15061228.
3. Scholz, J.; Weil, P.P.; Pembaur, D.; Koukou, G.; Aydin, M.; Hauert, D.; Postberg, J.; Kreppel, F.; Hagedorn, C. An Adenoviral Vector as a Versatile Tool for Delivery and Expression of miRNAs. Viruses 2022, 14, 1–18, doi:10.3390/v14091952.
4. Candia, A.J.; Garcia Fallit, M.; Peña Agudelo, J.A.; Pérez Küper, M.; Gonzalez, N.; Moreno Ayala, M.A.; De Simone, E.; Giampaoli, C.; Casares, N.; Seilicovich, A.; et al. Targeting FOXP3 Tumor-Intrinsic Effects Using Adenoviral Vectors in Experimental Breast Cancer. Viruses 2023, 15, 1813, doi:10.3390/v15091813.
5. Fallit, M.; Pidre, M.L.; Asad, A.S.; Peña Agudelo, J.A.; Vera, M.B.; Nicola Candia, A.J.; Sagripanti, S.B.; Pérez Kuper, M.; Amorós Morales, L.C.; Marchesini, A.; et al. Evaluation of Baculoviruses as Gene Therapy Vectors for Brain Cancer. Viruses 2023, 15, 1–21, doi:10.3390/v15030608.
6. Bentes, G.A.; Lanzarini, N.M.; Guimarães, J.R.; Heinemann, M.B.; Volotão, E. de M.; da Silva, A. dos S.; Heneine, L.G.D.; de Oliveira, J.M.; Pinto, M.A. Production and Evaluation of Chicken Egg Yolk Immunoglobulin (IgY) against Human and Simian Rotaviruses. Viruses 2022, 14, 1–10, doi:10.3390/v14091995.
7. Sabino, J.S.; Amorim, M.R.; de Souza, W.M.; Marega, L.F.; Mofatto, L.S.; Toledo-Teixeira, D.A.; Forato, J.; Stabeli, R.G.; Costa, M.L.; Spilki, F.R.; et al. Clearance of Persistent SARS-CoV-2 RNA Detection in a NFκB-Deficient Patient in Association with the Ingestion of Human Breast Milk: A Case Report. Viruses 2022, 14, doi:10.3390/v14051042.
8. Segura, E.; Ayoub, P.; Hart, K.; Kohn, D. Gene therapy for β-Hemoglobinopathies: From Discovery to Clinical Trials. Viruses 2023, 15, doi:10.3390/v15030713.

Virus vectors encoding foreign antigens offer several advantages for vaccine development as they stimulate cellular immune responses and facilitate efficient cellular uptake due to the active infection they cause. In this sense, the work presented by Moor et al. (contribution 1) describes the development of a lumpy skin disease virus-vectored vaccine used against SARS-CoV-2 (LSDV-SARS2-S, N), expressing a SARS-CoV-2 spike and nucleocapsid protein. The vaccine induced high titers of neutralizing antibodies in mice and hamsters, less viral effects and reduced viral RNA copies when hamsters were infected with SARS-CoV-2, showing promise as an effective vaccine against SARS-CoV-2.

In contrast to currently used lipid nanoparticle formulations, the capsid surface of viral vectors can be genetically and chemically modified, allowing us to specifically deliver small RNAs to certain organs or tissues. Gene therapy treatments based on adeno-associated virus (AAV) vectors have many advantages, and are gaining approval for commercial use [12]. There are, however, still some challenges they face; one of these is the presence of DNA contaminants from plasmid fragments obtained during the production process or host genomic DNA. Although most of these contaminants are thought to be innocuous, the formation of contaminants such as replication-competent (rc) AAV are clear concerns. There is a lack of information on the formation of these rcAAVs, and in this context, Yip et al. (contribution 2) used single-molecule real-time sequencing (SMRT) technology to analyze rcAAVs amplified via preparation of an AAV vector. Their findings suggest that rcAAVs are produced via random recombination, provided that all components for replication and packaging are present, thereby providing great insight into how rcAAVs are formed.

There are other disadvantages limiting the widespread use of Ad vectors, such as the powerful stimulation of adaptive and innate immune responses. However, there are some genetic and chemical modifications that may be performed to overcome this obstacle, as a shielding strategy to protect Ad vectors from interaction, recognition, and sequestration by the immune system. In the present work by Sholz et al. (contribution 3), the authors show the functionality of an adenoviral (Ad)-based miRNA vector in the efficient delivery and expression of a high level of miRNAs. The authors also demonstrate that the designed vector platform allowed the accurate dosing of the delivered miRNAs in contrast to non-viral systems, and allowed the downregulation of multiple endogenous target RNAs. Another interesting observation were synergistic effects on the efficiency of transduction using two different Ad-vectors, a finding that may be taken into consideration for the experimental design of further assays.

In another study presented in this SI, that of Candia et al. (contribution 4), presents the development of an adenoviral vector (Ad.P60) encoding a regulatory T cell master transcription factor, Foxp3 (Forkhead box P3) binder peptide (P60). The authors showed that this vector efficiently transduced breast tumor cells, decreased the migration and viability of the cells, and improved the cytotoxic response to cisplatin, a chemotherapy drug. In addition, the use of Ad P60 in affected mice induced both a delay in tumor growth and inhibition of lung metastases. This study showed that P60, which inhibits Foxp3, facilitates the efficacy of immunotherapeutic strategies alongside improving the response to chemotherapy in breast cancer, and also showed that adenoviral vectors are an excellent choice to facilitate these treatments by allowing the delivery of such peptides and contributing to the treatment of affected patients.

One concern regarding the use of adenoviral vectors (Adv) is the pre-existing immunity presented by humans. To overcome this issue, Fallit et al. (contribution 5) have studied baculoviral vectors (BV) for brain cancer therapy. The authors used mice to show that BVs transduced glioma cells and astrocytes with no apparent neurotoxicity, providing evidence that BV vectors are potential tools for the delivery of therapeutic genes to the brain.

Bentes et al. (contribution 6) developed a method for the production and purification of polyclonal antibodies against rotavirus group A, as an alternative for the prevention and treatment of rotavirus disease.

Sabino et al. (contribution 7) present a very interesting case report of an immunodeficient patient chronically infected with the SARS-CoV-2 Gamma variant. After treatment with breast milk from a vaccinated mother, patient showed improvements in their symptoms, and tested negative for SARS-CoV-2. These results suggest that IgA and IgG antibodies present in breast milk may be useful to treat persistent SARS-CoV-2 infection in immunocompromised patients.

Segura et al. (contribution 8) contributed to this SI with a review focusing on the adversities faced and achievements made during decades of studies on β-globin gene research. The authors highlight the importance of viral biotechnology and emerging technological advancements in β-globin vector research and successful gene therapies for β-thalassemia and sickle cell disease.

## 3. Concluding Remarks

The manuscripts featured in this Special Issue encompass studies on a wide range of virus applications, offering invaluable knowledge to science.

As Guest Editors of this Special Issue “The Application of Viruses to Biotechnology, 2022”, we would like to thank to all the authors for submitting such interesting contributions. It has been a pleasure to read and learn from their works. We would also like to thank the reviewers for their valuable comments on the manuscripts, as well as the Editorial Office for all their support.

Due to rapid advances in this exciting field of research, we are now launching the third volume of this Special Issue—“The Application of Viruses to Biotechnology 3.0”, which will collate further interesting studies on novel applications of viruses.
